# Predicting the recombination potential of severe acute respiratory syndrome coronavirus 2 and Middle East respiratory syndrome coronavirus

**DOI:** 10.1099/jgv.0.001491

**Published:** 2020-09-09

**Authors:** Arinjay Banerjee, Andrew C. Doxey, Benjamin J.-M. Tremblay, Michael J. Mansfield, Sonu Subudhi, Jeremy A. Hirota, Matthew S. Miller, Andrew G. McArthur, Samira Mubareka, Karen Mossman

**Affiliations:** ^1^​ Department of Pathology and Molecular Medicine, McMaster University, Hamilton, Ontario, L8S 4K1, Canada; ^2^​ Michael G. DeGroote Institute for Infectious Disease Research, McMaster University, Hamilton, Ontario, L8S 4K1, Canada; ^3^​ Department of Biology, University of Waterloo, Waterloo, Ontario, N2L 3G1, Canada; ^4^​ Division of Respirology, Department of Medicine, McMaster University, Hamilton, Ontario, L8S 4K1, Canada; ^5^​ Genomics and Regulatory Systems Unit, Okinawa Institute of Science and Technology Graduate University, Onna, Okinawa 904-0495, Japan; ^6^​ Gastrointestinal Unit and Liver Center, Massachusetts General Hospital, Harvard Medical School, Harvard University, Boston, MA 02114, USA; ^7^​ Department of Biochemistry and Biomedical Science, McMaster University, Hamilton, Ontario, L8S 4K1, Canada; ^8^​ Sunnybrook Health Sciences Centre, Toronto, Ontario, M4N 3M5, Canada; ^9^​ Department of Laboratory Medicine and Pathobiology, University of Toronto, Toronto, Ontario, M5S 1A8, Canada

**Keywords:** coronavirus, SARS-CoV-2, MERS-CoV, recombination, emergence, predictions

## Abstract

Severe acute respiratory syndrome coronavirus 2 (SARS-CoV-2) recently emerged to cause widespread infections in humans. SARS-CoV-2 infections have been reported in the Kingdom of Saudi Arabia, where Middle East respiratory syndrome coronavirus (MERS-CoV) causes seasonal outbreaks with a case fatality rate of ~37 %. Here we show that there exists a theoretical possibility of future recombination events between SARS-CoV-2 and MERS-CoV RNA. Through computational analyses, we have identified homologous genomic regions within the ORF1ab and S genes that could facilitate recombination, and have analysed co-expression patterns of the cellular receptors for SARS-CoV-2 and MERS-CoV, ACE2 and DPP4, respectively, to identify human anatomical sites that could facilitate co-infection. Furthermore, we have investigated the likely susceptibility of various animal species to MERS-CoV and SARS-CoV-2 infection by comparing known virus spike protein–receptor interacting residues. In conclusion, we suggest that a recombination between SARS-CoV-2 and MERS-CoV RNA is possible and urge public health laboratories in high-risk areas to develop diagnostic capability for the detection of recombined coronaviruses in patient samples.

## Introduction

A new coronavirus (CoV), severe acute respiratory syndrome coronavirus 2 (SARS-CoV-2), emerged in December 2019 [1]. SARS-CoV-2 evolved from CoVs found in bats [1, 2], and closely related viruses have been recently discovered in pangolins [3]. Bats have also been identified as reservoirs of several other CoVs [4], including CoVs capable of infecting human cells [5]. SARS-CoV-2 belongs to the genus *Betacoronavirus* (beta-CoV), which also includes severe acute respiratory syndrome coronavirus (SARS-CoV), which caused the epidemic of 2003–04 [6] and the Middle East respiratory syndrome coronavirus (MERS-CoV), which emerged in 2012 and continues to cause seasonal outbreaks in the Kingdom of Saudi Arabia (KSA) [7, 8]. MERS-CoV causes severe lower respiratory tract infections and has a case fatality rate of ~37 % [9], which is much higher than the reported 1.38 % mortality rate for SARS-CoV-2 in China [10].

The emergence of SARS-CoV-2 and the ongoing circulation of MERS-CoV in KSA raises an important question: can genetic material from the two viruses recombine? Although the case fatality rate of SARS-CoV-2 is lower than those for MERS-CoV and SARS-CoV [9, 10], it has infected more individuals and in a much shorter period of time. To detect any potential recombinant virus, it is imperative to identify high-risk geographical areas for SARS-CoV-2 and MERS-CoV co-infections and develop diagnostic assays for the surveillance of recombinant viruses in these areas.

Earlier studies have demonstrated that coronavirus genomes can recombine [11, 12], including within the coding region of the receptor-binding domain (RBD) of the spike (S) protein, which interacts with host cell receptors [13]. Recombination events may give rise to novel CoVs with enhanced or reduced ability to cause disease. In addition, since neutralizing antibodies specific for particular CoVs are raised against the spike protein [14], the emergence of a novel recombinant CoV may bypass existing CoV immunity in a population. Recombination between species within the genus *Betacoronavirus* have been described, such as those between canine respiratory CoV and bovine CoV [15], human CoVs OC43 and HKU1 [16, 17], and HKU1 and murine hepatitis virus (MHV) [18]. SARS-CoV was a product of recombination and MERS-CoV has already demonstrated ample capacity for recombination between lineages [19–21]. Thus, there is a risk that a recombinant CoV may escape SARS-CoV-2 immunity within the global population.

In this study, we have analysed the possibility of recombination between SARS-CoV-2 and MERS-CoV using bioinformatic analysis. We have identified homologous regions within SARS-CoV-2 and MERS-CoV genomes that may support recombination. We have discussed possible outcomes of a recombination event, along with the molecular properties of a potential recombinant virus. In addition, we have identified human tissues that may accommodate a recombination event based on receptor distribution for the two viruses. Our analyses indicate that although recombination between SARS-CoV-2 and MERS-CoV is possible, it is unlikely to happen in the respiratory tract and is more likely to occur in the gastrointestinal system, where both receptors are strongly co-expressed. Furthermore, as we enter periods of high global MERS-CoV activity (April to December) [22], we highlight the need for public health laboratories in high-risk areas to develop diagnostic capability for the detection of recombined CoVs in patient samples.

## Methods

### Alignment of SARS-CoV-2 and MERS-CoV genomes and analysis of similarity

SARS-CoV-2 (NCBI accession: NC_045512.2) and MERS-CoV (NCBI accession: NC_019843.3) genome sequences and annotations were downloaded from GenBank. Alignment and visualization of homologous regions were performed using the ‘FindSynteny’ function of the DECIPHER Bioconductor R package [23], using the settings ‘maxSep=200’ and ‘maxGap=600’. Sliding window analysis was done by first aligning the two genomes with clustal Omega [24] using default settings. From this alignment the percentage identity between the two sequences was calculated with a sliding window of 30 nucleotides using the R statistical programming environment [25]. Structural visualization of the SARS-CoV-2 RNA-dependent RNA polymerase (PDB ID: 6M71 [26]) was performed using PyMol (pymol.org).

### Co-expression analysis

ACE2 (ensembl ID: ENSG00000130234.10) and DPP4 (ensembl ID: ENSG00000197635.9) expression levels across human tissues were extracted on 26 April 2020 from GTEx Analysis Release v8 (dbGaP Accession phs000424.v8.p2) available at https://gtexportal.org/ [27, 28]. Microarray data from GEO dataset GSE75214 were retrieved and adjusted for batch correction using COMBAT [29] as implemented in the Bioconductor R package sva. Microarray analysis involved analysis of distinct samples and *P*-values were computed using two-tailed tests with cor.test() in R. Co-expression analysis was performed in R, and the top 100 co-expressed genes were identified based on Pearson correlation *r* scores. Function enrichment analysis was performed by analysis of the top 100 ACE2-correlated genes using enrichR with default settings [30].

### Phylogenetic and comparative analysis of virus receptors

Sets of orthologous ACE2 and DPP4 proteins were retrieved from the National Center for Biotechnology Information (NCBI) Gene database on 22 April 2020. The original set of ACE2 orthologues consisted of 300 sequences, DPP4 consisted of 235 sequences, and the union of the 2 sets consisted of 218 sequences. These sequences were aligned using the l-INS-i algorithm of the MAFFT package (v7.407) [31, 32], and a maximum-likelihood tree was estimated using RAxML (v8.2.4) with four gamma-distributed categories of rate heterogeneity, and automatic evolutionary model selection (v8.2.4; the JTT model was automatically selected for both the ACE2 and DPP4 alignments) [33, 34]. Phylogenies were visualized using Jalview (v2.11.0) [35] and the ape R package (v5.3) [36]. Bootstrapping was performed the number of times required to converge the bootstrap support signal using the extended majority rule consensus bootstrapping algorithm (autoMRE) implemented in RAxML, converging after 250 and 200 bootstraps for the ACE2 and DPP4 phylogenies, respectively. Each ACE2 and DPP4 orthologue was also directly compared to its human orthologue using the needle program of the EMBOSS package (v6.5.7.0) [37, 38]. The percentage identities for these alignments are available in Table S1 (available in the online version of this article). Human receptor residues within 3.5 Å of the virus–receptor complex structures were retrieved using PyMol (v2.3.5; https://pymol.org/2/) and the conservation of these homologous sites was visualized with ggseqlogo (v0.1) in R [39]. All raw data files and scripts have been deposited on Github and can be accessed at https://github.com/mjmansfi/BanerjeeEtAl_CoV-recomb.

## Results

### SARS-CoV-2 and MERS-CoV genomic sequences contain regions that can facilitate homologous recombination

**Fig. 1.**
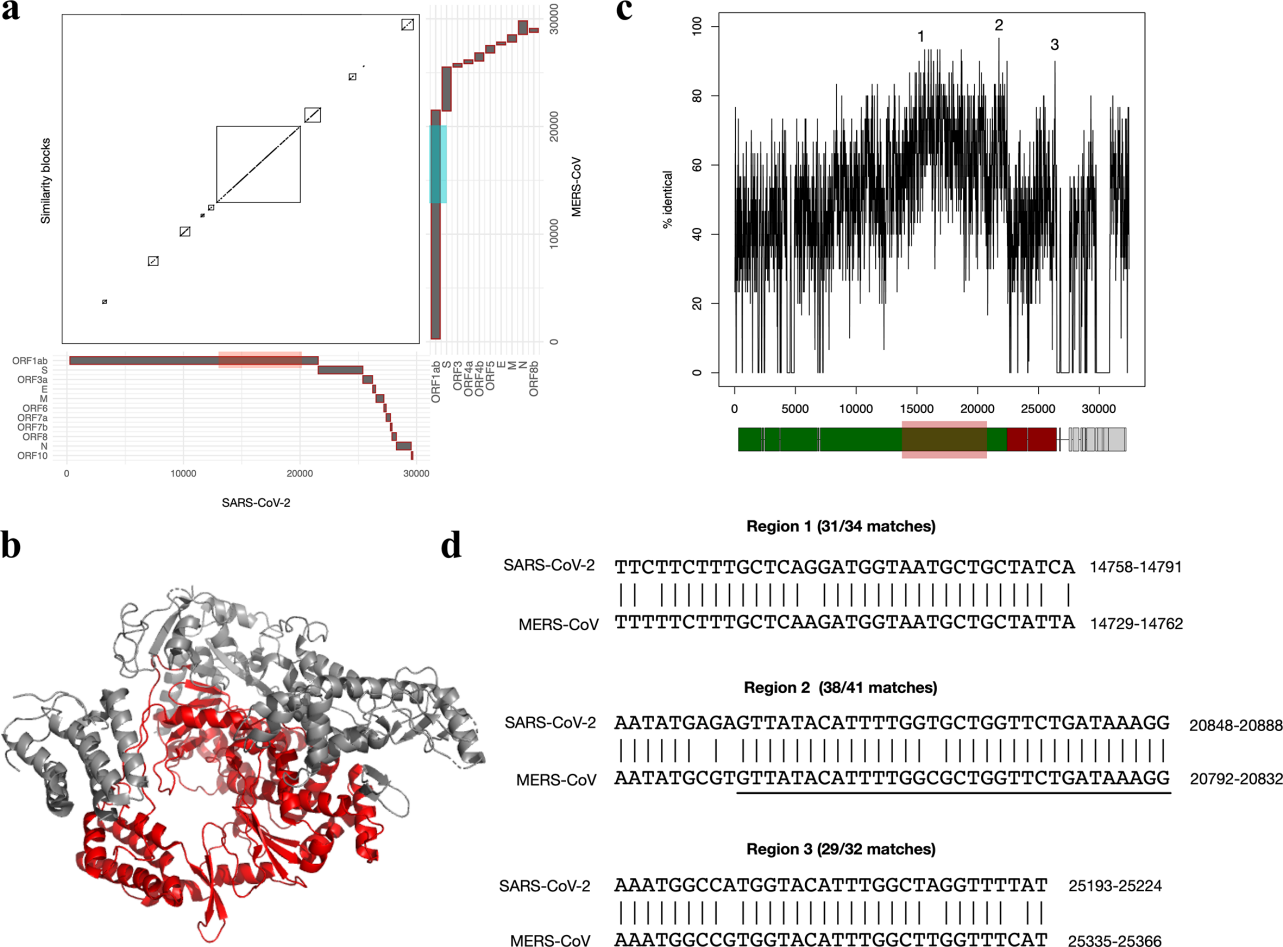
Recombination potential of SARS-CoV-2 and MERS-CoV. (a) Pairwise alignment of the reference genomes of SARS-CoV-2 and MERS-CoV. The gene locations for both viral genomes are plotted on the *x-* and *y*-axes. Detected syntenic blocks are shown in the plot. The largest synteny block detected occurs between region 12 944–19 922 in SARS-CoV-2 and region 12909–19875 in MERS-CoV. (b) Structure of SARS-CoV-2 RNA-dependent RNA polymerase (PDB ID: 6M71 [26]), with the region of similarity with MERS-CoV from (a) highlighted in red. (c) Sliding window analysis of SARS-CoV-2 : MERS-CoV genome alignment, displaying the percentage identity of all 30 length nucleotide segments. The axis numbering corresponds to alignment position. (d) Alignments of three selected regions of sequence similarity between SARS-CoV-2 and MERS-CoV. Alignments from (c) were extended in the 5′ and 3′ direction if additional matching positions were present. Region 2 includes a 32 bp segment with only one mismatch (underlined). The axis numbering corresponds to the position within the SARS-CoV-2 genome.

During CoV replication and transcription, viral RNA forms double-stranded RNA intermediates [40–42], facilitating the possibility of homologous recombination [43, 44]. To examine the genomic potential for recombination between SARS-CoV-2 and MERS-CoV, we aligned the reference genomes of SARS-CoV-2 and MERS-CoV, and identified syntenic regions of high pairwise sequence similarity. The largest detected block of similarity occurs in the region between 12 944 and 19 922 bp in SARS-CoV-2, and region between 12 909 and 19 875 bp in MERS-CoV (Fig. 1a). This region corresponds to the majority of the C-terminal portion of the ORF1ab protein, encoding the viral RNA polymerase (Fig. 1b). The overall nucleotide sequence identity across this entire region is relatively low at 64.73 %, which decreases the probability of homologous recombination. However, homologous recombination events in mammalian cells can occur at low frequencies in regions with as few as 14 bp in common [45]. Therefore, we searched for shorter segments of high sequence identity by performing a sliding window analysis, which plots the percentage identity of all 30 base pair segments (Fig. 1c). Consistent with the analysis in Fig. 1a, the most similar segments between the two genomes occur within an overlapping region of ORF1ab located between 13 798 and 20 788 bp. Examples of high-scoring pairs within ORF1ab (labelled regions 1 and 2) are shown in Fig. 1d; after extension, these regions have sequence identities of 31/34 bp (91 %) and 38/41 bp (93 %). Notably, region 2 includes a 32 bp segment with only one mismatch (underlined in Fig. 1d). Outside of ORF1ab, there are very few regions of similarity, with the exception of one segment (labelled region 3; sequence identity of 29/32 bp) that occurs in a 3′ region of the S gene encoding the S2 domain (Fig. 1d).

Although our analysis identified high-identity segments containing few mismatches, even smaller segments with 100 % identity also exist, including the 20 bp segment 5′-TTTAAATATTGGGATCAGAC-3′ (region 14299–14318 bp in SARS-CoV-2 and region 14 270–14 289 bp in MERS-CoV). Furthermore, we must consider the possibility of future mutations in these genomic locations that could increase the identity and potential for homologous recombination. Ultimately, this analysis suggests that although there is limited sequence identity across the full SARS-CoV-2 and MERS-CoV genomes, there are segments with sufficient sequence similarity to support potential recombination mediated by homologous base pairing.

### Human tissues co-express receptors for SARS-CoV-2 and MERS-CoV

**Fig. 2.**
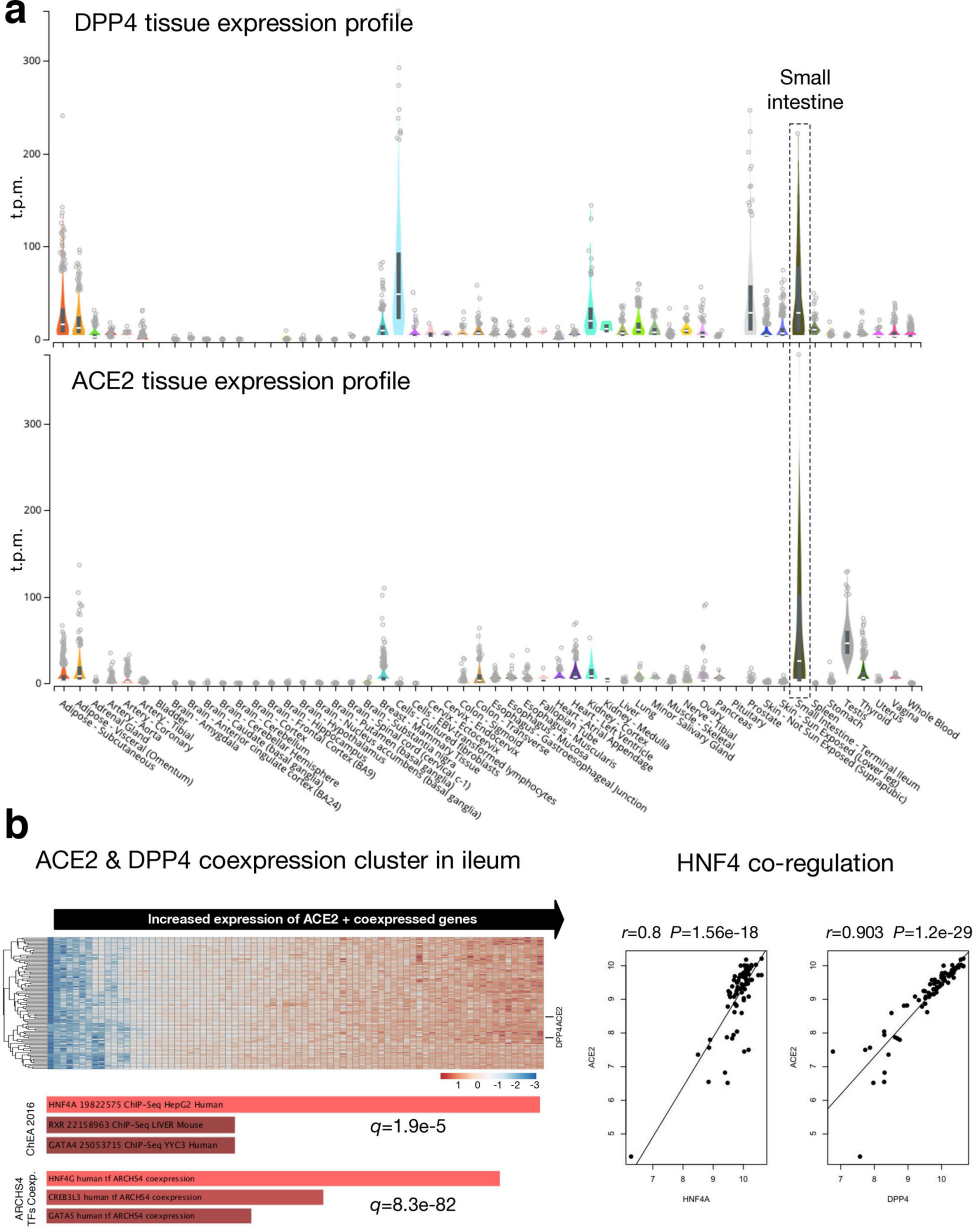
Co-expression profile of angiotensin-converting enzyme 2 (ACE2) and dipeptidyl peptidase 4 (DPP4). (a) RNA-seq based expression profile of ACE2 and DPP4 across 54 tissues. Expression data are derived from the GTEx database [27]. ‘Small intestine – terminal ileum’ is highlighted as a key tissue of interest since ACE2 and DPP4 are co-expressed at high relative expression levels compared to other tissues. The centre line denotes per-sample median expression level. (b) Analysis of ACE2 and DPP4 co-expression in GEO dataset GSE75214, including microarray expression profiles of ileum samples from healthy individuals, and individuals with inflammatory bowel disease and Crohn’s disease [28]. Left: a heatmap of the top 100 ACE2 co-expressed genes. For the heatmap, the *x*-axis includes all samples in the microarray dataset, while the *y*-axis includes represented genes. Values in each cell represent gene expression levels. Horizontal lines on the right indicate ACE2 and DPP4. Right: ACE2/HNF4A and ACE2/DPP4 co-regulation. t.p.m., transcripts per million.

For a recombination event to occur, SARS-CoV-2 and MERS-CoV would need to infect the same cell, which will facilitate close proximity interaction of RNA from the two viruses. As identified recently, SARS-CoV-2 uses angiotensin-converting enzyme 2 (ACE2) as a receptor to enter mammalian cells [46]. MERS-CoV uses dipeptidyl peptidase 4 (DPP4) as a cell receptor [47]. To identify tissues with ACE2 and DPP4 co-expression, we analysed publicly available gene expression data from the GTEx portal, which include tissue samples collected from ~1000 individuals across 54 non-diseased tissues [27]. Based on normalized expression levels (transcripts per million, t.p.m.) from RNA-seq experiments, we identified that ACE2 and DPP4 were co-expressed in numerous tissues including adipose tissue, mammary tissue, colon, kidney and small intestine (terminal ileum) (Fig. 2a). Among these tissues, we detected the highest levels of ACE2 and DPP4 co-expression in the small intestine.

In our analysis, we did not detect high levels of co-expression of ACE2 and DPP4 in human lung tissue; however, both SARS-CoV-2 and MERS-CoV cause respiratory infections and associated disease symptoms in humans [48, 49]. Thus, the possibility of recombination in human lung tissue or cells within the respiratory tract cannot be ruled out. As ongoing research identifies susceptible cell populations within the human respiratory tract at single-cell resolution, we shall be able to pinpoint cell types within the upper and lower respiratory tracts that may facilitate co-infections with SARS-CoV-2 and MERS-CoV.

To further examine possible co-expression of ACE2 and DPP4 in the ileum, we explored a microarray dataset (GEO accession GSE75214) of ileum samples from healthy individuals, as well as individuals with inflammatory bowel disease and Crohn’s disease [28] (Fig. 2b, left). ACE2 and DPP4 show a significant pattern of co-expression across these samples (Pearson correlation *r*=0.9, *P*=1.2e-29) (Fig. 2b, right). Furthermore, DPP4 is among the top 100 ACE2-correlated genes in this dataset. Function enrichment analysis of the top 100 ACE2 co-expressed genes revealed a significant association with the hepatocyte nuclear factor 4 (HNF4) family of transcription factors, including HNF4A/G (Fig. 2b, left and right). Therefore, HNF4A- or HNF4G-dependent gene expression patterns in the gastrointestinal system, which are regulated by host–microbiome interactions during inflammation [50] appear to drive upregulation of both ACE2 and DPP4 and may therefore be an important factor underlying the potential for SARS-CoV-2 and MERS-CoV gastrointestinal co-infection and recombination.

### Risk of SARS-CoV-2 and MERS-CoV recombination in other mammals

**Fig. 3.**
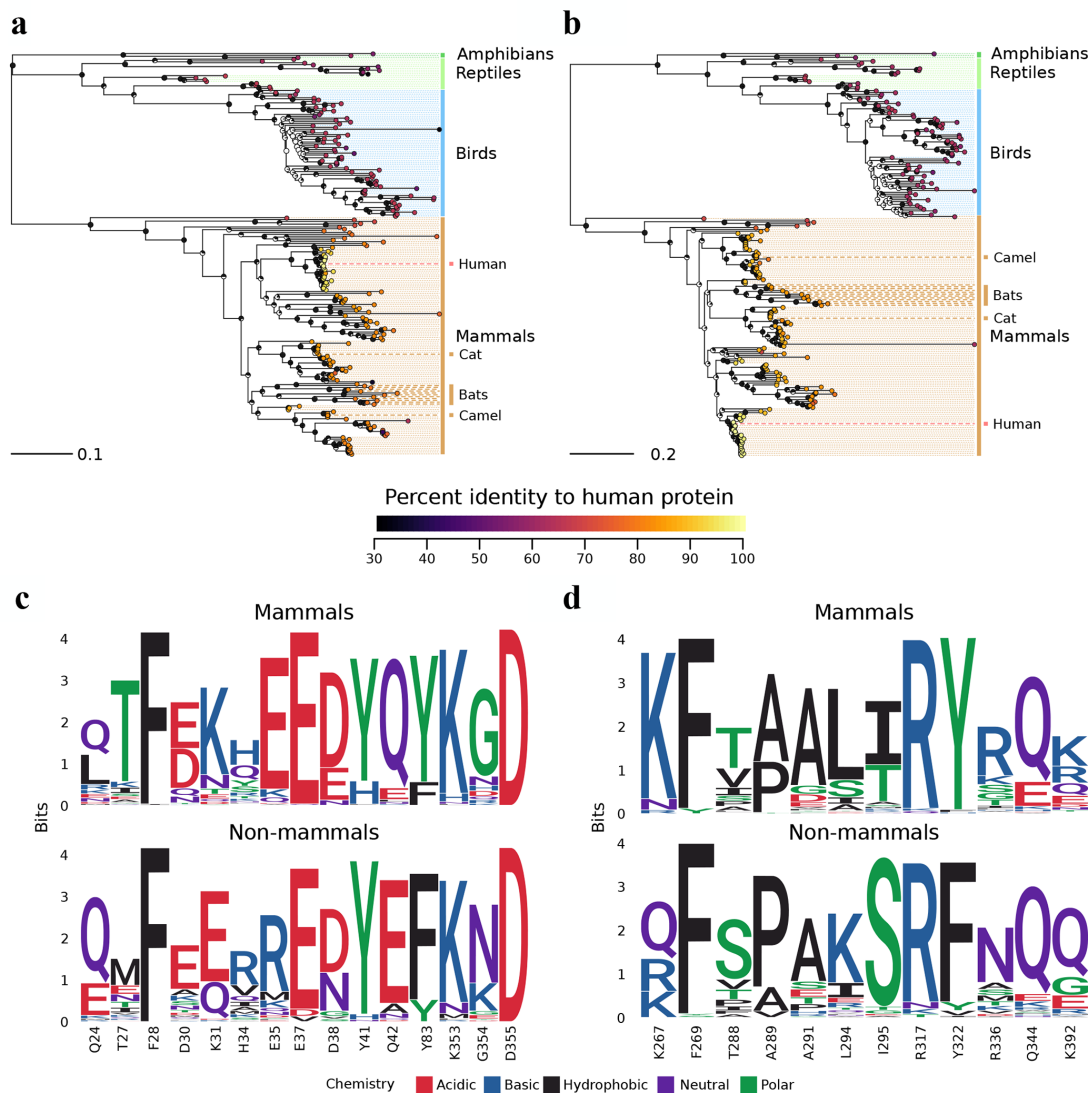
Evolutionary relationships of ACE2 and DPP4 proteins across various animal species. (a, b) Phylogenetic relationships of ACE2 and DPP4 orthologues across various animal species. Species that are known or suspected to be infected by either virus are indicated, namely cats, bats and the dromedary camel. Each tip in the tree represents a sequence from a different animal species, and the tips are annotated by coloured circles representing percentage identity to their human orthologue. Node labels represent bootstrap support as a fraction of a circle, where a greater proportion of the circle being black indicates a greater proportion of bootstrap support. The full list of sequences and their identities are available in Table S1. (c, d) Conservation of residues within the virus spike–receptor interfaces for ACE2 and DPP4 across animal species.

To identify animals that may be susceptible to both viruses and thereby represent potential reservoirs where recombination might occur between SARS-CoV-2 and MERS-CoV, we performed a phylogenetic analysis of their receptor proteins across amphibians, reptiles, birds and mammals (Fig. 3a, b). In general, ACE2 orthologues from other animals are less similar to human ACE2 (74.8 % amino acid identify, +/−12.4 %, minimum 31.3 %; Table S1) than DPP4 orthologues (77.0 % amino acid identity, +/−13.8 %, minimum 50.1 %; Table S1). We also examined patterns of sequence conservation among residues that participate in the human–viral spike protein interfaces from complexed structures of ACE2 with SARS-CoV-2 and DPP4 with MERS-CoV, respectively [51, 52] (Fig. 3c, d). In general, these residues are more strongly conserved between humans and mammals compared to more distantly related animals. Notably, this includes the dromedary camel, which is identical at 12/15 ACE2 and 9/12 DPP4 virus-contacting residues (yielding full-length alignments with human ACE2 at 83.6 % and human DPP4 at 85.5 % identity; Table S1). Based on our analysis, we believe that recombination is most likely to happen in susceptible mammals with receptors that are more similar to human ACE2 and DPP4. Thus, it is important to identify whether known reservoirs of MERS-CoV, such as dromedary camels, can also be infected with SARS-CoV-2. Recent studies have identified that cats and dogs can be productively infected with SARS-CoV-2 [53, 54]. Thus, it may also be useful to identify if cats and dogs can be infected with MERS-CoV.

## Discussion

SARS-CoV-2 and MERS-CoV outbreaks are currently occurring simultaneously in KSA, which raises concerns about recombination between two highly pathogenic CoVs. Multiple studies have shown that CoVs can recombine [13, 15, 16, 18, 20, 21, 55–57], but there are currently no studies that have predicted recombination events between SARS-CoV-2 and MERS-CoV. Experimental validation of potential recombination between SARS-CoV-2 and MERS-CoV poses clear gain-of-function risks that must be carefully weighed. Our computational analyses shed light on possible recombination breakpoints and the likely tissues that may support co-infection and recombination between SARS-CoV-2 and MERS-CoV. In our analysis, we have identified several genomic regions, including segments predominantly within the *ORF1ab* gene, that could support homologous recombination between SARS-CoV-2 and MERS-CoV (Fig. 1). In theory, a recombination event involving a breakpoint at this region could be capable of producing two recombinant viruses [1], a virus with a genome consisting of ORF1ab from SARS-CoV-2+MERS CoV along with remaining ORFs from SARS-CoV-2 [2] and a virus with a genome consisting of ORF1ab from SARS-CoV-2+MERS CoV along with remaining ORFs from MERS-CoV. CoV *ORF1ab* codes for non-structural proteins (nsps) that are essential in forming the replicase and transcriptional complexes, along with proteases that are required to cleave polyproteins pp1a and pp1ab into respective nsps [42]. A critical step in the generation of a replication-competent recombinant virus is the compatibility of proteins from the two viruses. The compatibility of nsps in ORF1a and ORF1ab is critical to facilitate the replication and transcription of the viral genome [58]. Compatibility between structural proteins is important for virus packaging, maturation and egress [42]. Mini-replicon assays can be performed using nsps from SARS-CoV-2 and MERS-CoV to determine what combinations, if any, are compatible [59].

The recombination frequency of CoVs can be as high as 25 % for the entire genome [55]. Currently, we do not know if RNA recombination of SARS-CoV-2 and MERS-CoV can produce replication-competent viruses, but studies with murine beta-CoVs, such as MHV, have demonstrated the ability of CoV RNA to recombine and produce replication-competent viruses [60]. Furthermore, recombination sites at the 5′-end of murine CoV RNA have been identified, along with the isolation of recombinant CoVs containing single and double crossovers in 5′-end genes [61]. Keck *et al.* also identified a third class of recombinants that contained crossovers within the leader sequence located at the 5′-end of the genome, along with one recombinant that contained a triple crossover [61]. Homologous CoV RNA recombination was also demonstrated in a study where MHV-A59 and defective interfering (DI) RNA containing MHV-RI spike gene were shown to recombine to produce a series of recombinant MHV genomes with chimeric S gene [62]. In our analysis, we were able to identify short homologous segments with high sequence identity within SARS-CoV-2 and MERS-CoV ORF1ab that may facilitate homologous recombination (Fig. 1). Multiple studies have proposed that RNA recombination is a frequent event during MHV replication, but similar recombination events remain to be identified in SARS-CoV-2. Furthermore, recombination events in mammalian cells can occur at low frequencies with as little as 14 bp in common [45]. In our analyses, we detected three segments >14 bp that aligned with high sequence identity between SARS-CoV-2 and MERS-CoV genomes (Fig. 1).

In addition to homologous recombination, CoVs are also capable of non-homologous recombination [56, 57] due to the ability of CoV RNA-dependent RNA polymerase (RdRp) to switch RNA strands during sub-genomic RNA synthesis [13, 57, 63]. During synthesis of sub-genomic RNA, CoV RdRp stops at transcriptional regulatory sequences (TRSs) that are at the beginning of each structural and accessory gene and switches its template to continue amplifying the leader sequence at the 5′-end to generate full-length sub-genomic RNA [64, 65]. This ability to switch templates may allow CoV RdRp to switch between the genomes of SARS-CoV-2 and MERS-CoV to generate chimeric sub-genomic RNAs. Emerging data suggest that RNA from beta-CoVs, such as MHV, MERS-CoV and SARS-CoV-2, perform extensive recombination in culture and these recombination events are facilitated by the CoV proofreading non-structural protein, nsp14 [66]. In theory, RNA recombination may produce hybrid SARS-CoV-2 and MERS-CoV sub-genomic RNAs and subsequently mRNA transcripts and proteins. More experimental work is required to identify whether these chimeric proteins will be more or less functional compared to their native counterparts in their respective viruses.

The potential ability of SARS-CoV-2 and MERS-CoV genomes to recombine raises many concerns, including the role of accessory proteins in modulating human immune responses. MERS-CoV accessory proteins are very efficient in counteracting human innate antiviral responses by blocking interferon (IFN) production and signalling [67–70]. A recent study identified that SARS-CoV-2 can inhibit antiviral IFN production; however, interestingly, downstream expression of IFN stimulated genes (ISGs) were observed in cells that were experimentally infected with SARS-CoV-2 [71]. Thus, any recombination between SARS-CoV-2 and MERS-CoV genomes may generate viruses with unpredictable pathogenic potential. Furthermore, CoVs may also recombine with other RNA viruses in rare cases, such as the recently reported recombination between a bat coronavirus (Ro-BatCoV GCCDC1) and a segment of the p10 gene from a bat orthoreovirus [72]. Thus, in addition to the possible recombination potential of SARS-CoV-2 and MERS-CoV that we have analysed here, other possibilities and opportunities for recombination of SARS-CoV-2 and other RNA viruses, including seasonal beta-coronaviruses, such as HKU1 and OC43, may exist.

RNA recombination between SARS-CoV-2 and MERS-CoV genomes may produce chimeric proteins, which in turn may affect the efficacy of drug interactions. Thus, it is important to identify potential recombination breakpoints and develop pan-CoV drugs that are effective in inhibiting the replication of diverse CoVs.

To facilitate RNA recombination, SARS-CoV-2 and MERS-CoV infections need to coincide in common human tissues and cells. A recent study identified a strong correlation between ACE2 and DPP4 expression using data from single-cell RNA sequencing [73]. In our analysis, we identified tissues including human kidneys and intestinal ileum as sites of ACE2 and DPP4 co-expression (Fig. 2). MERS-CoV is known to infect kidneys to cause kidney damage (acute renal failure) and multiple organ dysfunction in acute cases [74–76]. Recent data suggest that SARS-CoV-2 can infect kidney cells and tissues [77]. Emerging data from experimental infection of primary human intestinal epithelial cells suggest that SARS-CoV-2 is capable of infecting and generating replication-competent viruses in these cells [78]. In our analysis, we did not detect high levels of co-expression of ACE2 and DPP4 in human lung tissue; however, both SARS-CoV-2 and MERS-CoV cause respiratory infections in humans [48, 49]. Thus, the possibility of recombination in human lung tissue or cells within the respiratory tract cannot be ruled out. Ongoing research will shed more light on the full range of SARS-CoV-2 receptors and co-receptors, optimal levels of receptor expression required to facilitate CoV entry, and respiratory tract tissues and cell types that are susceptible to SARS-CoV-2 and MERS-CoV.

According to our analyses of GTEx data, the highest levels of ACE2 and DPP4 co-expression appear to be in the small intestine (Fig. 2a). This ileal co-expression pattern was confirmed by our independent analysis of ileum microarray samples (Fig. 2b). In the ileum, ACE2 and DPP4 display a significant pattern of co-expression, which appears to be driven by the HNF4 gene regulatory network [50]. SARS-CoV-2 has been detected and isolated from human faeces [79, 80]. Furthermore, a recent study demonstrated that SARS-CoV-2 can replicate in human gut enterocytes [81]. MERS-CoV has also been detected in human stool specimens. One study reported the detection of MERS-CoV RNA in 14.6 % of stool samples from infected individuals [82]. In addition, primary intestinal epithelial cells, small intestine explants and intestinal organoids have been demonstrated to support MERS-CoV replication [83]. Indeed, a co-infection of MERS-CoV and SARS-CoV-2 in renal or small intestinal tissues, especially the kidney cortex and ileum, may facilitate RNA recombination between the two viruses.

In addition to humans, other mammals may also pose a risk of co-infection with SARS-CoV-2 and MERS-CoV, thus providing RNA from the two viruses with an environment to recombine. Our analyses indicate that the patterns of similarity of ACE2 and DPP4 to their human orthologue are largely consistent with speciation (Fig. 3a, b). However, even distantly related animals can possess conserved residues necessary for virus–receptor interaction. A recent study identified that ferrets and domestic cats were susceptible to SARS-CoV-2, while dogs, pigs, ducks and chickens were not efficiently infected by the virus [53]. Similarly, MERS-CoV infects and replicates in dromedary camels [84–86]. Thus, there is a need to determine if camelids are susceptible to SARS-CoV-2 and whether cat species can support MERS-CoV replication. Further, the contacting residues for our structural comparisons (Fig. 3c, d) were identified within specific co-crystals, and these contacts may change in the context of variant host receptors or mutations within the virus. As SARS-CoV-2 spreads in the human population, it may produce more divergent viruses, and a new variant might facilitate an infection in a different animal host, or potentially even the use of a novel receptor, sparking the potential for recombination with known or yet unknown beta-CoVs. Exploratory studies are required to identify tissue level distribution and expression patterns of CoV receptors, including ACE2 and DPP4, in other mammalian species. Further studies are also required to determine orthologues of SARS-CoV-2 and MERS-CoV receptors in animals, along with functional studies to determine if cells from a wide range of animals can facilitate infection with these CoVs and their potential recombination.

Our analyses suggest that recombination between SARS-CoV-2 and MERS-CoV is possible in endemic areas that may facilitate co-infection. Thus, global public health experts and frontline physicians and diagnostic laboratories need to be prepared for such an occurrence. KSA has reported 2179 cases of COVID-19, with 29 deaths [87]. Between 1 December 2019 and 30 January 2020, there have been 19 cases of MERS-CoV, including 8 associated deaths [88]. As we enter periods of high global MERS-CoV activity (April to December) [22], it is imperative that surveillance programmes are capable of detecting co-infection and recombinant CoVs. There is a need to develop ancestrally reconstructed pan-CoV bait capture assays [89] to further expand upon the efforts of Li *et al.* [90] to capture and detect RNA from SARS-CoV-2/MERS-CoV recombinant viruses. Rapid capture and sequencing diagnostics will allow frontline diagnostic laboratories to enrich patient samples for SARS-CoV-2, MERS-CoV and any potential recombinant viruses. The bait sets can be deployed in high-risk areas to actively survey and monitor circulating SARS-CoV-2 and MERS-CoV variants or recombinants, or in response to molecular diagnosis of co-infection in individual patients. In addition, from a public health perspective, it would be strategic to separate COVID-19 and MERS patients in hospitals and perhaps have dedicated staff handling cases of each disease in high risk areas. Both SARS-CoV-2 and MERS-CoV have demonstrated their ability for nosocomial spread. Thus, broader public health awareness is necessary to manage intake of COVID-19 patients in high-risk MERS-CoV endemic areas. While continued and serious efforts to control the ongoing COVID-19 pandemic are necessary, we must also be prepared to identify and curb the spread of any antigenically novel SARS-CoV-2/MERS-CoV recombinants.

## Supplementary Data

Supplementary material 1
